# A Multi-Metric Approach to Investigate the Effects of Weather Conditions on the Demographic of a Terrestrial Mammal, the European Badger (*Meles meles*)

**DOI:** 10.1371/journal.pone.0068116

**Published:** 2013-07-11

**Authors:** Pierre Nouvellet, Chris Newman, Christina D. Buesching, David W. Macdonald

**Affiliations:** 1 Wildlife Conservation Research Unit, Department of Zoology, University of Oxford, The Recanati-Kaplan Centre, Tubney House, Tubney, Abingdon, Oxfordshire, United Kingdom; 2 Medical Research Council Centre for Outbreak Analysis and Modelling, Department of Infectious Disease Epidemiology, Imperial College London, London, United Kingdom; University of Sao Paulo, Brazil

## Abstract

Models capturing the full effects of weather conditions on animal populations are scarce. Here we decompose yearly temperature and rainfall into mean trends, yearly amplitude of change and residual variation, using daily records. We establish from multi-model inference procedures, based on 1125 life histories (from 1987 to 2008), that European badger (*Meles meles*) annual mortality and recruitment rates respond to changes in mean trends and to variability in proximate weather components. Variation in mean rainfall was by far the most influential predictor in our analysis. Juvenile survival and recruitment rates were highest at intermediate levels of mean rainfall, whereas low adult survival rates were associated with only the driest, and not the wettest, years. Both juvenile and adult survival rates also exhibited a range of tolerance for residual standard deviation around daily predicted temperature values, beyond which survival rates declined. Life-history parameters, annual routines and adaptive behavioural responses, which define the badgers’ climatic niche, thus appear to be predicated upon a bounded range of climatic conditions, which support optimal survival and recruitment dynamics. That variability in weather conditions is influential, in combination with mean climatic trends, on the vital rates of a generalist, wide ranging and K-selected medium-sized carnivore, has major implications for evolutionary ecology and conservation.

## Introduction

The difficulties of determining future climatic conditions present a major issue for both human societies and natural systems [1], prompting ecologists to consider how environmental variability might shape important patterns and processes in nature [2]–[4]. Our ability to predict the consequences of climate change requires that we fully understand how species may, or may not, be able to adapt to changing conditions over a range of temporal and spatial scales [5]. Individual fitness is linked to an optimal range of environmental conditions such that a species' climatic niche (see [6]) is a defining element in its evolution [7], [8]. For instance, there is imposed selection for a bounded range of temperature and humidity to which a species' physiology is adapted and its behaviour optimised [9] – where optimal weather conditions define a ‘Goldilocks zone’ [10], [11] outside of which more extreme conditions (e.g., too hot/cold; dry/wet) stress life-history optimality.

In the face of environmental change, such as more frequent episodes of extreme weather, species will attempt to adapt behaviourally, or evolve new physiological tolerances to cope with altered conditions, while vagile species may also move spatially to maintain existing physiological associations with the particular climates that define each species' climatic niche [12], [13]. Weather exceeding the tolerance limits of these conservative climatic niches [14], however, can destabilise the underlying selection pressures to which a species is exposed [15], [16], overwhelming evolutionary adaptation rates and behavioural flexibility [17]. Changes in climate averages, resulting from recent acceleration in climate change [18], [19] and departure from established regional weather patterns [20], [21], generate changes in selective pressures [20], [22], with detrimental impacts on both optimal phenotype [23] and biodiversity [24]. As a consequence, a major challenge is to better understand how population demographic parameters interact with weather patterns, in order to establish how species may respond to changes in climate averages [18] as well as changes in climate variability [20], [25].

Previously, we established the detailed metrics of European badger (*Meles meles*; Linnaeus 1758) population dynamics [26], and evidenced that these interact with climate according to long-term trends and seasonally sensitive periods [27], [28]. Johnson et al. [29] found that the annual difference in maximum and minimum temperature, recorded at 32 study sites across Europe, correlated consistently with both badger and sett (e.g., burrow system) densities, in both single-variable and multiple regressions; they concluded that regional badger densities are associated with seasonal amplitude (e.g., annual temperature range), or variables that covary with seasonality.

Our objective in this study was therefore to explore the effects of inter-annual variability from ‘typical’ mean conditions on key demographics. While this is of specific interest to badger ecology, as a temperate generalist carnivore the badger also exemplifies how a species with a broad range of bioclimatic niche tolerance [9] can be impinged by an under-investigated facet climate change, namely variability [25].

In the United Kingdom, badgers have no contemporary predators, thus, aside from disease [30], [31], there are few factors that could result in “top-down” population regulation (*sensu* [32]). Badgers also exhibit high spatial fidelity, invested in their setts, and are fundamentally contractionist, being slow to colonize vacant habitats [33].

We use data from our ongoing long-term population study of badgers at Wytham Woods, Oxfordshire, UK [26]–[28] – a Climate Change Network Long Term Environmental Research site (ILTER, www.ilternet.edu; [34]). We use a decomposition of yearly climatic conditions into annual mean temperature and rainfall, and also within-year amplitude and variability around the seasonal cycle. We then quantify the effects of variation in these different climatic components on juvenile and adult survival rates and recruitment [35], using capture-mark-recapture and multi-model inference procedures. This approach allowed us to test the hypothesis that a range of optimal climatic influences might be in operation – specifically that demographic rates may respond to non-linear (quadratic) components of climate (range extremes), and that the consequences of these interactions may be age-class dependent.

## Materials and Methods

### Study site and population

Wytham Woods is a 424 ha mixed semi-natural woodland site, 5 km north west of Oxford, UK (GPS ref: 51∶46:26N; 1∶19∶19W; Mean annual temperature 10.1°C; mean annual precipitation 644.8 mm; for details see [27], [34].

The resident badger population is discrete geographically with a stable range (see [26]). Studied continuously since the 1970 s [36], these badgers have been live-trapped and marked systematically three to four times per annum (each season) since 1987 with a high trapping efficiency (despite no pre-baiting, thus ensuring that all badgers receive non-influential levels of supplementary food). Excluding 1987 (1st study year), trapping efficiency for this population remained relatively constant throughout with a mean of 83.69% (SE  = 1.32%) badgers caught yearly (for details see [27]).

### Climate data: Means, Amplitudes and Standard Deviations

All demographic analyses were limited to 1987–2008. Temperature and rainfall figures were provided by the Radcliffe Meteorological Station, School of Geography, University of Oxford, within 6 km of the Wytham Woods research site. We used daily temperatures available from 1881 to 2008 inclusive and daily rainfalls from 1987 to 2008 inclusive. Although only ‘post 1986’ temperatures were used as a covariate in the demographic analyses, we use the full temperature dataset to establish long-term trends. This was the highest resolution of climatic variables that these data permitted, and the measure most pertinent to the badgers' micro-climate-related foraging success [37] and thermo-regulatory budgets while outside of subterranean burrow system [38].

For biological life-history relevance, we defined ‘year’, hereafter, to start from 1^st^ March, which corresponds approximately with the peak date of parturition. As mean climatic trends are composed of simultaneous effects on the frequency and amplitude of variation [39], we derived metrics that reflected proximate climatic conditions for each year.

#### Temperature

**Figure 1:**
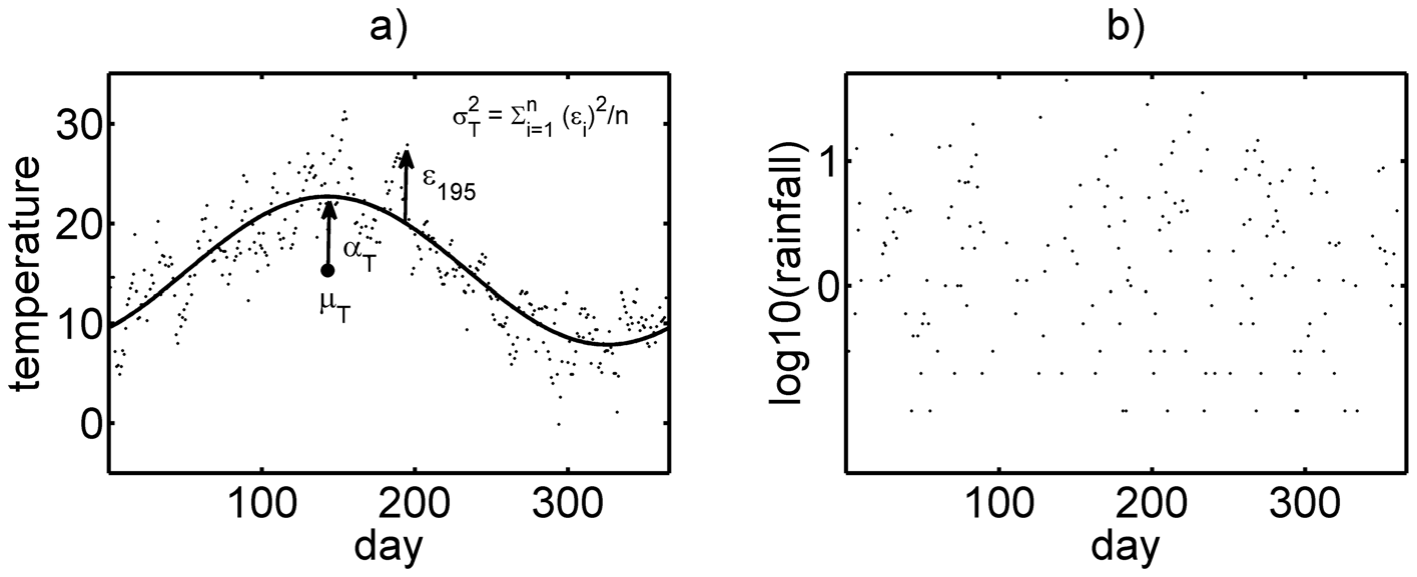
Representative plot of (a) Daily temperature in the Oxford study area over one year from 1^st^ March 2000; demonstrating a clear sinusoidal seasonal trend. The solid line represents the effect of yearly mean temperature, 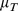, combined with temperature amplitude, 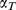, in 2000. Some variance around the predicted temperatures (the solid line) is evident, defined as 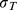
(b) Daily rainfallover the Oxford study area over one year from 1^st^ March 2000. No seasonal trends were apparent. The year 2000 was taken as an example and while there is inter-annual variation, other years followed the same general temperature and rainfall patterns.

As daily temperatures, 1881–2008, showed an oscillating, seasonal, pattern, as expected from the changing angle of incidence of the sun at the latitude of the study area (Figure 1a), and given the sinusoidal nature of the variation of annual temperatures, we characterized yearly temperature with a trigonometric (cosine) function, to include 3 metrics: i) Mean (average) temperature for that year: 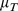; ii) Amplitude of seasonal temperature changes: 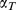 – representing the maximum temperature deviation (in summer or winter) from the average yearly temperature; and iii) Residual standard deviation around the daily predicted temperature values, 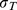
- providing a measure of the yearly temperature variability.

In our model, the temperature *T* on day *d* of year *y* is thus characterized as:
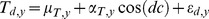
where: 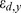
represents the residual associated with year ‘y’ and day ‘d’, and each 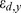 value is assumed to follow a normal distribution with mean 0 and standard deviation 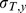, and *c* is a constant (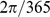) such that the cycle has a period of one year.

We estimated 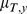, and 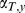, for each year, for the period 1881 to 2008, using a regression procedure (function ‘*regress*’ from Matlab, The MathWorks). The sum of squared residuals over each year allowed us to derive the yearly temperature variance, 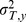 and thus 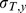; effectively similar to employing a ‘sinusoidal model’ [25], [40].

#### Rainfall

No within-year seasonal trends were evident (Figure 1b). This permitted rainfall, within any given year, to be characterised simply by the daily mean rainfall, 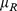, and the coefficient of variation 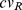. These two indices were calculated from 1987 to 2008.We used the coefficient of variation in rainfall rather than standard deviation, as the latter is highly correlated with the mean rainfall. This approach is consistent with numerous climatic studies (e.g., [41], [42]).

### Influence of Weather Metrics on Survival and Recruitment

Throughout our study we use a demographic history file documenting the capture events of 1125 individual badgers, caught between 1 and 33 times, between 1987 and 2008. We have established previously that badger population dynamics can be modelled successfully using stage-classified matrix models [26], where the survival of sexually mature and immature individuals (juveniles [cubs to <1 year old] and adults [≥1]: *φ_j_* and *φ_a_*), and fecundity (*F*), are parameterised. We estimated survival and recruitment sequentially, using the mark-recapture framework (implemented in the MARK program, ([43], version 6.0). We used the Pradel method to estimate recruitment, but not survival rates, as this method would not have allowed survival to be age-dependent. The sequential method we present allowed us to explore potential differences in juvenile, compared to adult, survival rate responses to climate. Variation in capture rates was modelled as time and age-class (i.e., juvenile and adult) dependent in all models [44], such that capture probabilities varied between trapping sessions, years and age classes.

#### Survival rate

Estimates of juvenile and adult survival rates, using the ‘*Cormack-Jolly-Seber* ’ (CJS) model [45], [46], were used to construct a primary model of survival rates, 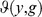 (survival, 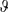, depends upon year, *y*, and age class, *g*). This model was checked for goodness of fit using bootstrap methods, allowing approximation of a corrected variance inflation factor (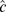). As intervals between trapping sessions (between two and seven months) were not constant throughout the study, we defined the time intervals between trapping events manually to derive yearly survival values. In all models presented we employed a *logit* transformation for the estimation of survival rates (being probabilities).

We then re-estimated survival rates, allowing the *logit* of survival estimates to follow a linear relationship with the standardized weather metrics (within the MARK progam). We stress that the survival rates, and their relationship with weather metrics, were re-evaluated simultaneously for each model. Predictor variables were standardised to enable direct comparison of the parameter estimates (i.e. we computed the Z-scores), where the notation 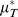 refers to the standardized value of 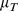. Models varied in term of which weather metrics they included, as predictors and were compared using a multi-model inference procedure [47].

From a biological standpoint, there was no *a priori* basis to assume linearity between the *logit* of survival rates and weather metrics (i.e, more plausibly we conceive of an optimal temperature associated with a peak survival rate; see [48]). Consequently, in all models presented, a quadratic term was used as well as a linear term. The significances of the estimates of each coefficient were assessed from 95% confidence intervals, with reference to any overlap with zero. Weather conditions might also affect juvenile and adult survival differently [28]. To account for this, we constructed models *with* and also *without* interaction terms (accounting for *differential* and *similar* relationships between age-classes' survival and weather metrics).

In multi-model inference, when Akaike predictor weights are compared, it is important that all variables are represented equally in the analysis. Given that five standardized weather metrics were derived for each year (Temperature: mean, amplitude and residual standard deviation; Rainfall: mean and residual standard deviation), we were able to construct 32 models without interaction i.e., 1 model with no climatic covariate, 5 models with one climatic covariate, and 10 models with two, 10 models with three, 5 models with four, and 1 model with five climatic covariates). Additionally, 31 models (each model with weather metric(s)) were constructed including an interaction term(s). We therefore evaluated 63 models, where juvenile and adult survival rates were estimated allowing, in 62 instances, the *logit* of these rates to be similarly, or differentially, linked to weather metrics by a linear and a quadratic component.

For each model, we derived the *QAICc* (*AIC* corrected for small sample size and adjusted for over-dispersion), which we used to rank the support for each model (a lower value indicating stronger model support); and also the Akaike weight for each model [47]. Each provided a different estimation of the coefficient(s) 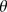
('s), linking the five weather metrics (and their squared values) to the *logit* of survival rate.

We applied model averaging to derive estimates of the 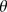
's associated with each model and their confidence intervals. Model averaging takes account of uncertainty in model selection by calculating the mean value for a coefficient of interest through averaging its value over all models in the candidate model set containing the coefficient of interest, weighted by normalised *AIC* weights [47]. Confidence intervals were based on estimated unconditional variance [47], accounting for two variance components: the conditional sampling variance associated with each – per model, and the variation associated with model selection uncertainty.

The ‘Relative Influence’ of a weather metric was defined as the sum of Akaike weights for all models including the predictor variable [47]. The predictor variable with the largest sum was inferred to be the most influential; the variable with the smallest sum was inferred to be the least influential predictor.

#### Recruitment

We performed separate analyses for survival and recruitment rates. Recruitment was estimated as a gross measure of the number of offspring entering the population records each year per individual (including juveniles), using the Pradel method in MARK (i.e., ‘Pradel survival and recruitment’ option) and a *log* link function.

We constructed 32 models predicting recruitment, *f*, as a function of standardized climate variables in the preceding year. Recruitment was constrained to a non-zero value only for the first trapping per annum following March, approximating the time when offspring (cubs) are born. Values for survival rates and capture probabilities were fixed to predetermined values, estimated using a CJS model with survival rates constant between age classes, but dependent upon year and annual capture rates [44].

## Results

### Weather metrics

#### Temperature

Mean temperature, 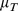, increased with year (*b* = 0.01 with *F*_126_ = 30.5, *p*<0.001, *R^2^* = 0.20), equivalent to a rise of 2°C since 1881 (see Text S1). No significant long-term trends were detected in seasonal amplitude, (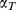), (*b* = 0.001 with *F*_126_ = 0.36, *p* = 0.56, *R^2^* = 0.003) (see Text S1), nor in yearly residual standard deviation, 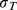 (*b* = −4 10^−3^ with *F*_126_ = 3.10, *p* = 0.09, *R^2^* = 0.02). An analysis of the correlations between temperature metrics is presented in Text S1.

#### Rainfall

Annual mean rainfall, 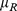, increased with time (*b* = 0.07 with *F*_20_ = 4.6, *p*<0.04, *R^2^* = 0.20), but no significant long-term trend in the coefficient of variation of rainfall, 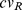, was found (*b* = 0.01 with *F*_20_ = 0.18, *p* = 0.68, *R^2^* = 0.01). We present an analysis of the correlations between rainfall metrics in Text S1.

### Influence of weather metrics on life-history parameters

Mean adult survival rate (0.81, SE 0.01) was higher than mean juvenile survival rate (0.67, SE 0.03), which concurs with previous studies [26], [28]. These estimates were derived from a model where survival rates were constants across years, irrespective of climatic conditions. In a similar constant model, recruitment across the study period averaged 0.76 (SE 0.01).

#### Survival rate

All 63 models were ranked according to their relative statistical support (see Table S1). Models that included interaction terms with age ranked highest (Table S1). The Relative Influence (sum of Akaike weights) of models with interactions was 0.66, compared to a Relative Influence of 0.33 for models without interaction, evidencing that weather conditions affected adult and cub survival differently. The Delta Akaike of the model including no weather metrics was around 9 (see Table S1), indicating weather metrics had a major influence on badger survival rate.

**Figure 2:**
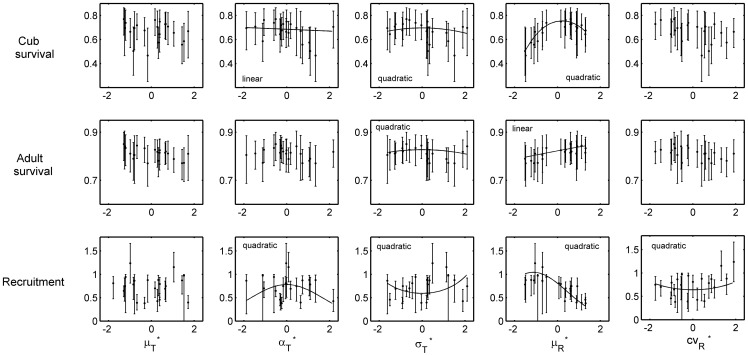
Survival rate estimates, for juveniles and adults, and recruitment rate as a function of climate metrics, with 95% confidence intervals (error bars, based on model averaging). The solid curve represents the statistically significant link between life-history parameters and climate metrics, for which we indicate whether the linear or quadratic (or both) component(s) was (were) significant. Importantly, a significant linear component *does not* imply a straight line in the representation shown, as the relationship is defined as linear within a logistic transformation (for survival rates), or within a log transformation (for recruitment).

Mean daily rainfall was the most influential predictor; its Relative Influence was more than three times higher than that of any other predictor (Table 1). For adults (Figure 2 and Table 1), the association of 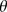
with 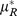
was significant and positive, but the relationship with 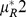 was not significant (the 95% CI overlapped with 0). Over the range of mean daily rainfall observed during the study period, adult survival was therefore lowest in the driest years.

**Table 1:** Model averaging for the parameters that link the survival rate of juvenile and adult badgers to standardized weather metrics within the logistic model.

Weather metric	Relative Influence	Juvenile Survival		Adult survival	
		θ	95% CI	θ	95% CI
*μ* ^*^ *_T_*	0.299	−0.035	−0.111, 0.041	−0.052	−0.121, 0.018
*μ* ^*^ *_T_* ^2^		−0.010	−0.041, 0.020	−0.001	−0.027, 0.025
*α* ^*^ *_T_*	0.131	−0.035*	−0.063, −0.007	0.006	−0.005, 0.017
α^*^ *_T_* ^2^		0.005	−0.009, 0.019	−0.002	−0.009, 0.005
σ^*^ *_T_*	0.277	−0.019	−0.059, 0.022	0.005	−0.019, 0.030
*σ* ^*^ *_T_* ^2^		−0.049*	−0.096, −0.001	−0.029*	−0.057, −0.002
*μ* ^*^ *_R_*	0.934	0.162*	0.061, 0.263	0.115*	0.043, 0.187
*μ* ^*^ *_R_* ^2^		−0.369*	−0.525, −0.213	−0.067	−0.165, 0.030
*cv* ^*^ *_R_*	0.155	0.019	−0.007, 0.046	−0.007	−0.019, 0.005
*cv* ^*^ *_R_* ^2^		0.019	−0.006, 0.044	−0.004	−0.015, 0.007

The Relative Influence of each metric (based on Akaike weights) is presented along with the model−averaged estimated values of their coefficients, 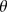
's, with confidence intervals, based on estimated unconditional variances. An ‘*’ was added where the estimated coefficient differs statistically from zero (based on 95% confidence intervals).

For juveniles (Figure 2 and Table 1), the association of 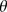
's with 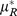 was significant and positive, but significant and negative for 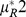. The strong influence of this 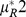 effect (Figure 2, Table 1) indicated that for juveniles an optimum survival rate was associated with intermediate rainfall.

The amplitude and variability of daily temperature had much less influence (Table 1 and Figure 2). For both adults and juveniles (Figure 2 and Table 1) the associations of 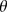
's with 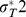 were negative, however confidence intervals bordered 0, indicating an effect bordering significance. Thus both adult and juvenile survival rates were overall greater when variability in temperature was closer to its mean value over the study period. For Juveniles (Figure 2 and Table 1), the association of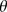 with 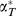 was negative, but confidence intervals again bordered 0 indicating only weak significance. Nevertheless, this gives limited support for juvenile survival being greater during years with lower amplitude of temperature change between winter-summer.

#### Recruitment

Again, all 32 models were compared and ranked according to their statistical support (see Table S2) revealing that mean rainfall (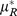) was the most influential covariate predicting recruitment (Table 2). Broadly, the Relative Influences of all weather metrics were high, and the most supported models contained many climatic covariates. We infer that recruitment is linked with weather more intricately than survival rates. The Delta Akaike of the model including no weather metrics was around 37 (see Table S2) demonstrating the importance of considering weather metrics when analysing badger recruitment.

**Table 2:** Model averaging for the parameters that link recruitment to standardized climate metrics with log transformation.

Weather metric	Relative Influence	Recruitment	
		θ	95% CI
*μ* ^*^ *_T_*	0.376	−0.013	−0.084, 0.058
*μ* ^*^ *_T_* ^2^		−0.007	−0.042, 0.029
*α* ^*^ *_T_*	0.922	−0.070	−0.149, 0.009
α^*^ *_T_* ^2^		−0.161*	−0.218, −0.104
*σ* ^*^ *_T_*	0.869	0.030	−0.024, 0.084
*σ* ^*^ *_T_* ^2^		0.118*	0.060, 0.175
*μ* ^*^ *_R_*	1.000	−0.454*	−0.544, −0.364
*μ* ^*^ *_R_* ^2^		−0.201*	−0.288, −0.114
*cv* ^*^ *_R_*	0.510	−0.023	−0.071, 0.025
*cv* ^*^ *_R_* ^2^		0.060*	0.011, 0.109

The Relative Influence of each metric (based on Akaike weights) is presented along with the model-averaged estimated values of their coefficients, 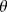
's, with confidence intervals, based on estimated unconditional variances. An ‘*’ was added where the estimated coefficients that differ statistically from zero (based on 95% confidence intervals not overlapping 0).

An optimal range of mean rainfall (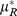), the most influential covariate, was apparent beyond which wetter conditions and, to a lesser extent, also drier conditions appeared to be detrimental for recruitment (Figure 2and Table 2); the association of 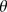
with 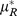 was strongly negative. In addition, but to a lesser extent, the association of 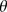 with 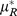
was also negative. The association of 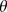 with 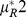 was also negative (Figure 2and Table 2), although not to the extent observed for 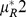. Any evidence, however, that there could be an association between optimal recruitment and intermediate amplitude of seasonal change (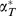) was less convincing.

The associations of 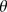
's with 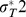 and 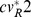 (Figure 2 and Table 2) were both positive, but with confidence intervals bordering 0, showing marginal evidence that was lowest for intermediate values of the standard deviation in temperature (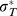) and of the coefficient of variation in rainfall (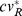).

## Discussion

By examining the influence of weather variability on survival and recruitment rates, our study contributes to a growing understanding of how mammals in general, and badgers in particular, respond to climatic conditions through looking at stressors of their climatic niche. In order to describe associations of climate with population dynamics, it is thus necessary to consider not just trends, or seasonal interactions, but also to quantify responses to (increasing) variability around long-term normative values (i.e., description of variability as well as mean trends). Our approach highlights the importance of considering the multiple facets of climate, and describes an effective way to decompose weather patterns for temperature and rainfall, which is both intuitive and easy to perform.

Our analyses highlight that juvenile and adult survival rates differed in their association with climate. Significant quadratic effects also lead us to conclude propose, more generally, that relationships between demographic parameters and climate may often not be linear. Indeed, we observed interactions that are both multi-component and differ with age class, highlighting some of the major challenges when attempting to link population dynamics and climate. Not least, this analysis serves to highlight the importance of long-term studies, and the value of detailed environmental data recording in order to conceive of effective responses to the challenges climate change poses on biodiversity.

Mean rainfall proved by far the most influential predictor, for badgers in this Oxfordshire study area, and was associated with different adult and juvenile survival rate responses. Juvenile survival and recruitment rates were highest at intermediate levels of mean rainfall (i.e., juveniles did poorly if it was not only too dry, but also if it was too wet – suggesting a ‘comfort zone’ or ‘Goldilocks zone’), whereas low adult survival was associated with only the driest, and not the wettest, years.

These findings enabled us to clarify the interaction between different, formerly paradoxical, facets of weather effects influencing badger population dynamics. In previous work, dry weather in spring/early summer has been linked to negative consequences for badger foraging success [27], believed to be caused by the highly weather dependent availability of earthworms [37], the predominant food resource for badgers in our study area [36], [28]. Thus, food deprivation due to dry spring conditions has been associated with restricted cub growth and lower survival rates [27], [49].

Simultaneously, dry spring weather has been established to ameliorate the otherwise deleterious consequences of disease and exposure to thermo-regulatory stress for badgers. Previous analyses have confirmed a link between high rainfall in spring, high levels of endo-parasitic infection, and reduced cub survival and recruitment [28, see also 50]. Badger cubs are highly susceptible to a coccidian, *Eimeria melis*, which has a 100% prevalence when cubs are first trapped in late May/early June [31]. Coccidiosis causes malabsorption of nutrients, diarrhoea, steatorrhoea, with subsequent fluid loss and electrolyte imbalance [51] – leading to morbidity and mortality. Weather front systems bringing wet conditions in the spring compound the stresses on vulnerable cubs by exposing them to additional hypothermic stress [52].

The pattern we observed in the present analyses affirmed both these positive and negative impacts of rainfall in a comprehensive manner. Adult survival, cub survival and recruitment were all lower during the driest years. Cub survival, and recruitment rate (the latter measure reflecting pre-trapping mortality in cubs, as an unknown proportion of cubs will inevitably die before we are able to trap them; see [27]), however, were both lower during the wettest years.

In response to variation from the long-term yearly temperature mean, the climatic niche sensitivity of both cubs and adults appears likely due to there being a range of tolerance for residual standard deviation around daily predicted temperature values, 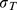 (Figure 2), although less so than for rainfall, as this effect bordered significance. We infer that predictable conditions seem to be important for optimal survival dynamics, allowing individual badgers to prospect risk most effectively [53] and optimise their annual routines [9].

The effect of mean rainfall was consistent for survival and recruitment rates. Similarly, the effect of standard deviation in temperature on survival rate was consistent for both juvenile and adults. The effects of the amplitude of seasonal change in temperature, and the effect of variability in temperature and rainfall on recruitment, however, had less support and caution should be used in interpretations. While an optimum in recruitment with intermediate temperature amplitude is conceivable, it is difficult to comprehend how intermediate variability in temperature and rainfall would lead to a minimum in recruitment. More data and analyses would be required to explore these relatively weaker interactions further.

As life-history strategies are shaped by environmental pressures, changes in the stability of optimal weather conditions expose the tolerance of populations to climatic stress and have fitness consequences [10], [54]. In terms of natural selection, species and clades tend to retain ecological traits over time through niche conservatism [55]. As a consequence, the adaptability of extant species is restricted by the physiological and behavioural repertoire inherited from their ancestors [15], limiting future possibilities through genetic limitations [14], or alternatively precipitating evolutionary change [56].

At the population level, however, not all the impacts of increased environmental variability appear to be negative [57]. Variability, rather than constancy, should be the focus of studies concerned with ecological resilience to changing conditions [13], [58]. Our multi-model inference procedures, linked with a quantitative decomposition of yearly climatic conditions, proved highly probative in this regard (see also [35]).

Interestingly, while we observed that mean temperature had risen significantly (2°C since 1881) at our study location, as had rainfall, we detected no long-term changes in seasonal amplitude or residual standard deviation for temperature or with respect to variation in rainfall patterns. This leads us to predict that while both juvenile and adult survival rates exhibited sensitivity to residual standard deviation in temperature, this component of climatic variability does not seem to impose any immediate threat to population viability. By contrast, absolute levels of mean rainfall proved critical (see also [28]) where crucially – although drought conditions are known to limit badger food supply – current trends toward wetter conditions would also compromise the population's tolerance.

The non-linear nature of the effects of these climatic components on population dynamics highlights the imperative of developing a better understanding of how projected increases in weather variability will operate [20], [59]. While rare species occupy a central place in biodiversity concerns, because they are the most prone to extinction [60], species that encounter a broader array of climatic conditions across their range are expected to have broader tolerances to climate change than restricted species [61]. Our evidence here that variation in weather conditions proved influential on the vital rates of a generalist, wide ranging and K-selected medium-sized carnivore, thus has major implications for the evolution of life-histories [4] and for conservation management in the face of uncertainty [62].

## Supporting Information

Table S1 **Statistical summary of the models linking survival rate to climate metrics.**
(PDF)

Table S2 **Statistical summary of the models linking recruitment to climate metrics.**
(PDF)

Text S1 **In this appendix, we provide analyses of correlation between weather metrics presented in the main text.**
(PDF)
