# Supplementary material for: A Multi-Metric Approach to Investigate the Effects of Weather Conditions on the Demographic of a Terrestrial Mammal, the European Badger (Meles meles)
Source: PLoS One. 2013 Jul 11;8(7):e68116. doi: 10.1371/journal.pone.0068116 (PMC3708947; doi:10.1371/journal.pone.0068116)
Supplement: Table S1 — Statistical summary of the models linking survival rate to climate metrics. (PDF) [file pone.0068116.s001.pdf]

Table S1

Model	QAICc	Δ QAICc	AICc Weights	Coefficient linking *adult* survival to specific climatic variables																			
				mT	SE	mT^2	SE	aT	SE	aT^2	SE	sT	SE	sT^2	SE	mR	SE	mR^2	SE	cvR	SE	cvR^2	SE
mR ,Int	10572	0.00	0.300													0.124	0.100	-0.016	0.128				
sT, mR, Int	10574	1.97	0.112									0.059	0.089	-0.104	0.081	0.177	0.115	-0.019	0.127				
mT, mR, NoInt	10574	2.50	0.086	-0.195	0.243	0.009	0.101									0.100	0.086	-0.180	0.108				
mR,NoInt	10575	2.95	0.069													0.125	0.080	-0.156	0.105				
mT, mR, Int	10575	3.17	0.061	-0.233	0.294	0.027	0.120									0.107	0.106	-0.030	0.131				
mR, cvR, Int	10575	3.67	0.048													0.075	0.119	-0.034	0.133	-0.046	0.092	-0.037	0.092
mT, sT, mR, NoInt	10576	3.93	0.042	-0.116	0.237	-0.026	0.102					-0.008	0.080	-0.112	0.072	0.112	0.102	-0.198	0.108				
sT, mR, NoInt	10576	4.12	0.038									-0.002	0.075	-0.113	0.069	0.150	0.093	-0.174	0.106				
aT, mR, Int	10576	4.50	0.032					0.048	0.105	-0.010	0.077					0.125	0.110	-0.028	0.145				
aT, mR, cvR, Int	10577	5.68	0.018					0.081	0.121	-0.039	0.087					0.056	0.135	-0.054	0.156	-0.083	0.106	-0.037	0.104
mT, sT, mR, Int	10578	5.81	0.016	-0.130	0.293	-0.015	0.123					0.050	0.096	-0.097	0.086	0.147	0.127	-0.026	0.132				
mT,NoInt	10578	6.12	0.014	-0.003	0.217	-0.088	0.089																
mT, mR, cvR, NoInt	10578	6.46	0.012	-0.208	0.247	0.012	0.103									0.120	0.101	-0.183	0.112	0.029	0.080	0.005	0.082
mT, aT, mR, Int	10578	6.48	0.012	-0.374	0.349	0.071	0.140	0.130	0.109	-0.051	0.079					0.095	0.123	-0.103	0.148				
all - mT ,Int	10578	6.62	0.011					0.128	0.142	0.001	0.092	-0.027	0.106	-0.114	0.091	0.079	0.169	-0.045	0.160	-0.107	0.116	-0.022	0.106
aT, mR, NoInt	10578	6.71	0.010					-0.040	0.090	-0.012	0.066					0.110	0.089	-0.150	0.119				
mT, mR, cvR, Int	10578	6.77	0.010	-0.217	0.300	0.015	0.122									0.087	0.122	-0.061	0.138	-0.002	0.090	-0.073	0.095
mR, cvR, NoInt	10579	6.94	0.009													0.121	0.098	-0.147	0.109	-0.008	0.082	0.028	0.080
mT, sT, NoInt	10579	7.35	0.008	-0.012	0.223	-0.084	0.095					-0.083	0.069	-0.055	0.068								
sT, mR, cvR, Int	10579	7.62	0.007									0.043	0.092	-0.111	0.086	0.128	0.136	-0.038	0.133	-0.052	0.092	-0.052	0.097
aT, sT, mR, NoInt	10579	7.64	0.007					-0.046	0.100	0.054	0.074	0.009	0.087	-0.140	0.079	0.190	0.108	-0.130	0.121				
aT ,Int	10579	7.79	0.006					0.006	0.101	-0.019	0.069												
aT, sT, mR, Int	10579	7.79	0.006					0.050	0.114	0.033	0.084	0.024	0.100	-0.127	0.091	0.196	0.131	-0.009	0.147				
all-aT,NoInt	10580	7.86	0.006	-0.115	0.242	-0.029	0.104					-0.013	0.082	-0.119	0.076	0.120	0.116	-0.211	0.113	0.016	0.081	-0.036	0.086
all-cvR,NoInt	10580	7.97	0.006	-0.157	0.279	-0.009	0.118	0.027	0.110	0.007	0.080	-0.024	0.099	-0.114	0.081	0.115	0.118	-0.203	0.126				
sT, mR, cvR, NoInt	10580	8.16	0.005									-0.004	0.076	-0.118	0.073	0.139	0.109	-0.177	0.109	-0.015	0.083	-0.013	0.083
cvR,NoInt	10580	8.27	0.005																	-0.120	0.061	0.077	0.079
mT, cvR, NoInt	10580	8.59	0.004	-0.043	0.217	-0.054	0.092													-0.074	0.066	0.058	0.083
all - sT ,Int	10580	8.66	0.004	-0.317	0.356	0.042	0.144	0.151	0.126	-0.096	0.097					0.025	0.144	-0.149	0.169	-0.070	0.101	-0.095	0.116
mT, aT, NoInt	10580	8.70	0.004	0.066	0.224	-0.109	0.090	-0.068	0.093	-0.031	0.060												
.,g),p(t,.)}	10580	8.72	0.004																				
cvR ,Int	10581	9.16	0.003																	-0.100	0.069	-0.011	0.090
sT,NoInt	10581	9.50	0.003									-0.103	0.065	-0.030	0.063								
mT, aT, Int	10581	9.73	0.002	-0.081	0.279	-0.064	0.107	0.075	0.102	-0.053	0.068												
all-sT,NoInt	10582	9.95	0.002	-0.168	0.281	-0.015	0.117	0.015	0.114	-0.065	0.085					0.065	0.125	-0.237	0.137	0.019	0.096	-0.033	0.097
aT,NoInt	10582	9.97	0.002					-0.134	0.092	0.010	0.062												
mT ,Int	10582	10.18	0.002	-0.010	0.263	-0.090	0.104																
mT, sT, cvR, NoInt	10582	10.47	0.002	-0.017	0.223	-0.069	0.097					-0.064	0.072	-0.065	0.071					-0.069	0.069	0.015	0.086
sT, cvR, NoInt	10582	10.50	0.002									-0.060	0.069	-0.054	0.066					-0.115	0.066	0.039	0.082
aT, mR, cvR, NoInt	10582	10.78	0.001					-0.048	0.108	-0.003	0.077					0.126	0.116	-0.141	0.126	0.015	0.098	0.012	0.088
all,Int	10583	11.03	0.001	-0.331	0.368	0.058	0.153	0.231	0.160	-0.045	0.102	-0.099	0.132	-0.093	0.095	-0.012	0.176	-0.135	0.171	-0.123	0.115	-0.062	0.120
all - cvR ,Int	10583	11.16	0.001	-0.312	0.349	0.051	0.144	0.139	0.124	-0.011	0.092	-0.024	0.116	-0.093	0.096	0.121	0.145	-0.084	0.152				
mT, aT, sT, NoInt	10583	11.37	0.001	0.004	0.254	-0.087	0.105	-0.028	0.106	0.014	0.072	-0.080	0.095	-0.057	0.071								
all - aT ,Int	10583	11.69	0.001	-0.113	0.298	-0.026	0.125					0.028	0.102	-0.105	0.092	0.120	0.148	-0.055	0.139	-0.015	0.091	-0.080	0.100
aT, cvR, Int	10583	11.69	0.001					0.097	0.116	-0.039	0.070									-0.137	0.082	-0.005	0.101
all-mT,NoInt	10583	11.71	0.001					-0.062	0.127	0.060	0.083	0.016	0.093	-0.142	0.081	0.212	0.150	-0.123	0.131	0.022	0.105	-0.003	0.088
sT ,Int	10583	11.73	0.001									-0.052	0.076	-0.031	0.075								
all,NoInt	10584	11.97	0.001	-0.133	0.293	-0.023	0.127	0.020	0.143	-0.007	0.093	-0.021	0.108	-0.116	0.082	0.107	0.155	-0.220	0.142	0.007	0.105	-0.035	0.098
aT, cvR, NoInt	10584	12.13	0.001					-0.046	0.106	-0.002	0.062									-0.100	0.074	0.058	0.089
mT, aT, cvR, NoInt	10584	12.25	0.001	0.004	0.242	-0.077	0.101	-0.022	0.110	-0.033	0.063									-0.059	0.079	0.027	0.094
aT, sT, NoInt	10584	12.63	0.001					-0.093	0.104	0.047	0.071	-0.086	0.083	-0.039	0.067								
mT, cvR, Int	10585	13.07	0.000	-0.045	0.269	-0.066	0.110													-0.049	0.075	-0.040	0.095
mT, sT, Int	10585	13.74	0.000	0.000	0.279	-0.097	0.114					-0.036	0.083	-0.055	0.083								
aT, sT, cvR, NoInt	10586	14.29	0.000					0.003	0.115	0.036	0.067	-0.076	0.079	-0.067	0.070					-0.119	0.077	0.051	0.088
aT, sT, Int	10586	14.37	0.000					0.061	0.115	0.016	0.077	-0.091	0.092	-0.048	0.078								
all-mR,NoInt	10586	14.41	0.000	-0.085	0.275	-0.040	0.118	0.042	0.126	0.017	0.072	-0.089	0.093	-0.072	0.072					-0.085	0.082	0.031	0.095
aT, sT, cvR, Int	10586	14.76	0.000					0.181	0.119	-0.002	0.072	-0.115	0.082	-0.146	0.079					-0.174	0.083	-0.045	0.097
sT, cvR, Int	10587	14.91	0.000									-0.020	0.081	-0.067	0.080					-0.113	0.075	-0.032	0.095
mT, aT, cvR, Int	10587	15.12	0.000	-0.130	0.303	-0.037	0.122	0.128	0.120	-0.071	0.073									-0.101	0.087	-0.049	0.109
mT, aT, sT, Int	10588	16.05	0.000	-0.175	0.324	-0.029	0.130	0.129	0.115	0.000	0.084	-0.103	0.112	-0.063	0.085								
all - mR ,Int	10590	18.05	0.000	-0.319	0.343	0.075	0.147	0.226	0.143	0.024	0.082	-0.177	0.108	-0.157	0.083					-0.167	0.090	-0.051	0.108
mT, sT, cvR, Int	10591	18.83	0.000	-0.010	0.282	-0.085	0.117					-0.034	0.088	-0.073	0.087					-0.057	0.079	-0.067	0.100
mT, aT, mR, NoInt	10640	68.47	0.000	-0.845	0.410	0.126	0.157	0.385	0.072	-0.338	0.053					-0.218	0.103	-0.564	0.118				

Model	Coefficient linking *juvenile* survival to specific climatic variables																			
	mT	SE	mT^2	SE	aT	SE	aT^2	SE	sT	SE	sT^2	SE	mR	SE	mR^2	SE	cvR	SE	cvR^2	SE
mR ,Int													0.173	0.141	-0.482	0.182				
sT, mR, Int									-0.137	0.131	-0.209	0.122	0.181	0.165	-0.563	0.190				
mT, mR, NoInt	-0.195	0.243	0.009	0.101									0.100	0.086	-0.180	0.108				
mR,NoInt													0.125	0.080	-0.156	0.105				
mT, mR, Int	-0.114	0.401	-0.050	0.172									0.128	0.147	-0.542	0.192				
mR, cvR, Int													0.299	0.173	-0.467	0.186	0.076	0.168	0.220	0.137
mT, sT, mR, NoInt	-0.116	0.237	-0.026	0.102					-0.008	0.080	-0.112	0.072	0.112	0.102	-0.198	0.108				
sT, mR, NoInt									-0.002	0.075	-0.113	0.069	0.150	0.093	-0.174	0.106				
aT, mR, Int					-0.235	0.155	-0.050	0.113					0.110	0.150	-0.438	0.200				
aT, mR, cvR, Int					-0.459	0.212	0.109	0.139					0.438	0.204	-0.369	0.203	0.387	0.211	0.145	0.145
mT, sT, mR, Int	-0.043	0.403	-0.078	0.178					-0.114	0.135	-0.224	0.125	0.135	0.168	-0.622	0.201				
mT,NoInt	-0.003	0.217	-0.088	0.089																
mT, mR, cvR, NoInt	-0.208	0.247	0.012	0.103									0.120	0.101	-0.183	0.112	0.029	0.080	0.005	0.082
mT, aT, mR, Int	0.113	0.430	-0.148	0.180	-0.197	0.161	-0.105	0.117					0.035	0.158	-0.556	0.217				
all - mT ,Int					-0.646	0.248	0.371	0.172	0.137	0.189	-0.448	0.198	0.887	0.330	-0.259	0.228	0.564	0.223	0.087	0.154
aT, mR, NoInt					-0.040	0.090	-0.012	0.066					0.110	0.089	-0.150	0.119				
mT, mR, cvR, Int	-0.337	0.432	0.054	0.188									0.291	0.181	-0.507	0.196	0.099	0.162	0.220	0.143
mR, cvR, NoInt													0.121	0.098	-0.147	0.109	-0.008	0.082	0.028	0.080
mT, sT, NoInt	-0.012	0.223	-0.084	0.095					-0.083	0.069	-0.055	0.068								
sT, mR, cvR, Int									-0.151	0.144	-0.159	0.129	0.290	0.187	-0.569	0.198	0.152	0.182	0.091	0.152
aT, sT, mR, NoInt					-0.046	0.100	0.054	0.074	0.009	0.087	-0.140	0.079	0.190	0.108	-0.130	0.121				
aT ,Int					-0.436	0.179	0.047	0.125												
aT, sT, mR, Int					-0.248	0.189	0.114	0.140	-0.004	0.171	-0.289	0.164	0.281	0.201	-0.425	0.213				
all-aT,NoInt	-0.115	0.242	-0.029	0.104					-0.013	0.082	-0.119	0.076	0.120	0.116	-0.211	0.113	0.016	0.081	-0.036	0.086
all-cvR,NoInt	-0.157	0.279	-0.009	0.118	0.027	0.110	0.007	0.080	-0.024	0.099	-0.114	0.081	0.115	0.118	-0.203	0.126				
sT, mR, cvR, NoInt									-0.004	0.076	-0.118	0.073	0.139	0.109	-0.177	0.109	-0.015	0.083	-0.013	0.083
cvR,NoInt																	-0.120	0.061	0.077	0.079
mT, cvR, NoInt	-0.043	0.217	-0.054	0.092													-0.074	0.066	0.058	0.083
all - sT ,Int	0.107	0.487	-0.130	0.215	-0.442	0.245	0.051	0.152					0.340	0.218	-0.470	0.224	0.383	0.223	0.089	0.170
mT, aT, NoInt	0.066	0.224	-0.109	0.090	-0.068	0.093	-0.031	0.060												
.,g),p(t,.)}																				
cvR ,Int																	-0.230	0.130	0.260	0.136
sT,NoInt									-0.103	0.065	-0.030	0.063								
mT, aT, Int	0.270	0.381	-0.166	0.164	-0.399	0.176	0.009	0.121												
all-sT,NoInt	-0.168	0.281	-0.015	0.117	0.015	0.114	-0.065	0.085					0.065	0.125	-0.237	0.137	0.019	0.096	-0.033	0.097
aT,NoInt					-0.134	0.092	0.010	0.062												
mT ,Int	0.000	0.362	-0.077	0.158																
mT, sT, cvR, NoInt	-0.017	0.223	-0.069	0.097					-0.064	0.072	-0.065	0.071					-0.069	0.069	0.015	0.086
sT, cvR, NoInt									-0.060	0.069	-0.054	0.066					-0.115	0.066	0.039	0.082
aT, mR, cvR, NoInt					-0.048	0.108	-0.003	0.077					0.126	0.116	-0.141	0.126	0.015	0.098	0.012	0.088
all,Int	0.168	0.530	-0.142	0.258	-0.625	0.272	0.292	0.188	0.127	0.197	-0.384	0.189	0.712	0.332	-0.345	0.245	0.518	0.219	0.063	0.171
all - cvR ,Int	0.154	0.465	-0.159	0.206	-0.243	0.205	0.053	0.146	0.021	0.184	-0.269	0.162	0.193	0.199	-0.523	0.227				
mT, aT, sT, NoInt	0.004	0.254	-0.087	0.105	-0.028	0.106	0.014	0.072	-0.080	0.095	-0.057	0.071								
all - aT ,Int	-0.168	0.426	-0.017	0.192					-0.123	0.147	-0.166	0.135	0.254	0.193	-0.608	0.209	0.147	0.170	0.091	0.158
aT, cvR, Int	-0.443	0.260	0.095	0.148													0.011	0.171	0.146	0.145
all-mT,NoInt					-0.062	0.127	0.060	0.083	0.016	0.093	-0.142	0.081	0.212	0.150	-0.123	0.131	0.022	0.105	-0.003	0.088
sT ,Int									-0.235	0.118	-0.051	0.109								
all,NoInt	-0.133	0.293	-0.023	0.127	0.020	0.143	-0.007	0.093	-0.021	0.108	-0.116	0.082	0.107	0.155	-0.220	0.142	0.007	0.105	-0.035	0.098
aT, cvR, NoInt					-0.046	0.106	-0.002	0.062									-0.100	0.074	0.058	0.089
mT, aT, cvR, NoInt	0.004	0.242	-0.077	0.101	-0.022	0.110	-0.033	0.063									-0.059	0.079	0.027	0.094
aT, sT, NoInt					-0.093	0.104	0.047	0.071	-0.086	0.083	-0.039	0.067								
mT, cvR, Int	-0.158	0.371	0.018	0.163													-0.196	0.133	0.256	0.138
mT, sT, Int	0.013	0.365	-0.060	0.164					-0.210	0.122	-0.079	0.115								
aT, sT, cvR, NoInt					0.003	0.115	0.036	0.067	-0.076	0.079	-0.067	0.070					-0.119	0.077	0.051	0.088
aT, sT, Int					-0.440	0.221	0.108	0.154	-0.045	0.168	-0.068	0.130								
all-mR,NoInt	-0.085	0.275	-0.040	0.118	0.042	0.126	0.017	0.072	-0.089	0.093	-0.072	0.072					-0.085	0.082	0.031	0.095
aT, sT, cvR, Int					-2.990	1.202	1.400	0.581	0.127	0.227	-0.125	0.176					0.636	0.268	-0.093	0.176
sT, cvR, Int									-0.160	0.129	-0.005	0.117					-0.164	0.140	0.205	0.145
mT, aT, cvR, Int	0.203	0.426	-0.137	0.186	-0.456	0.281	0.071	0.149									0.056	0.184	0.106	0.158
mT, aT, sT, Int	0.263	0.408	-0.161	0.180	-0.418	0.219	0.082	0.150	-0.025	0.176	-0.094	0.133								
all - mR ,Int	0.543	0.540	-0.115	0.225	-2.974	0.862	1.316	0.428	0.212	0.235	-0.088	0.158					0.620	0.222	-0.115	0.174
mT, sT, cvR, Int	-0.135	0.381	0.021	0.171					-0.153	0.132	-0.010	0.126					-0.139	0.141	0.202	0.151
mT, aT, mR, NoInt	-0.845	0.410	0.126	0.157	0.385	0.072	-0.338	0.053					-0.218	0.103	-0.564	0.118				

Table S1:

Statistical summary of the models linking survival rate to climate metrics.

The ‘model’ column provides a short model description, with: ‘T, R’ representing temperature and rainfall respectively; ‘m, a, s, cv’ the mean, amplitude of seasonal change, standard deviation and coefficient of variation (we used standardised variables); ‘Int , NoInt’ representing models with or without interaction terms (e.g. models with, or without, differential responses to climate metrics across age classes). The notation ‘.’ highlights the model including no climatic variables (e.g. estimating survival rates using a CMR model without covariates) while ‘all’ highlights the model with all climatic variables included (for simplicity, the model labelled ‘all –mT’ presents the model including all variables but the mean temperature and its quadratic component).

In the 3 next columns, the support for each model is presented, based on Akaike criterion and weight.

The furthest right column on the table presents the coefficient for each model linking the survival rate of juveniles and adults to specific climatic variables (we present the estimate for the linear and quadratic effect together with their standard error). When the model does not include an interaction term (noted ‘NoInt’), the coefficient(s) is the same for juvenile and adult survival.

Based on Akaike weight (w_i), the most supported model (μ_R) shows almost three time the support of the second ranked model (σ_T, μ_R). These two primary models both include an interaction, term highlighting the importance of considering differential relationships between climatic variables and the survival rates of juveniles and adults. This table is the basis of the model averaging, for which results are presented in Table 1 of the main text.
