# Supplementary material for: A Multi-Metric Approach to Investigate the Effects of Weather Conditions on the Demographic of a Terrestrial Mammal, the European Badger (Meles meles)
Source: PLoS One. 2013 Jul 11;8(7):e68116. doi: 10.1371/journal.pone.0068116 (PMC3708947; doi:10.1371/journal.pone.0068116)
Supplement: Table S2 — Statistical summary of the models linking recruitment to climate metrics. (PDF) [file pone.0068116.s002.pdf]

Table S3

Model	AICc	Delta AICc	AICc Weights	Coefficient linking *recruitment* to specific climatic variables																			
				mT	SE	mT^2	SE	aT	SE	aT^2	SE	sT	SE	sT^2	SE	mR	SE	mR^2	SE	cvR	SE	cvR^2	SE
all - mT	27307	0	0.301					-0.072	0.094	-0.179	0.064	0.036	0.062	0.151	0.063	-0.494	0.112	-0.219	0.100	-0.042	0.072	0.108	0.052
aT, sT, mR	27308	0	0.248					-0.108	0.075	-0.175	0.059	0.034	0.059	0.129	0.060	-0.457	0.082	-0.198	0.096				
all - cvR	27308	1	0.163	0.089	0.119	-0.075	0.056	-0.081	0.081	-0.201	0.061	0.051	0.063	0.114	0.061	-0.456	0.083	-0.227	0.098				
all	27309	2	0.119	-0.119	0.165	0.024	0.079	-0.018	0.104	-0.174	0.067	0.017	0.069	0.149	0.066	-0.479	0.113	-0.208	0.102	-0.050	0.075	0.132	0.072
aT, mR	27312	5	0.031					-0.114	0.070	-0.104	0.054					-0.372	0.062	-0.139	0.091				
mT, aT, mR	27312	5	0.028	0.023	0.111	-0.054	0.053	-0.054	0.077	-0.132	0.056					-0.372	0.065	-0.169	0.094				
all - aT	27312	5	0.024	-0.303	0.138	0.113	0.067					0.000	0.056	0.110	0.060	-0.342	0.082	-0.115	0.088	-0.029	0.058	0.197	0.064
mT, mR, cvR	27313	6	0.019	-0.241	0.131	0.074	0.062									-0.304	0.073	-0.093	0.086	-0.045	0.058	0.147	0.060
aT, mR, cvR	27313	6	0.017					-0.067	0.085	-0.114	0.060					-0.418	0.086	-0.149	0.093	-0.067	0.068	0.071	0.050
all - sT	27313	6	0.014	-0.122	0.148	0.015	0.070	-0.003	0.090	-0.116	0.065					-0.388	0.089	-0.145	0.098	-0.068	0.070	0.097	0.067
mR, cvR	27314	7	0.008													-0.355	0.069	-0.152	0.082	-0.057	0.053	0.093	0.049
sT, mR, cvR	27314	7	0.008									0.020	0.054	0.088	0.055	-0.370	0.076	-0.179	0.084	-0.031	0.055	0.124	0.052
mR	27315	7	0.007													-0.322	0.058	-0.168	0.078				
mT, mR	27315	8	0.007	-0.056	0.107	-0.014	0.050									-0.302	0.059	-0.136	0.081				
sT, mR	27316	9	0.003									-0.001	0.053	0.071	0.051	-0.353	0.067	-0.353	0.067				
mT, sT, mR	27317	10	0.002	-0.057	0.110	-0.008	0.052					0.007	0.056	0.049	0.054	-0.324	0.069	-0.145	0.082				
aT, sT, cvR	27325	17	0.000					-0.293	0.074	-0.003	0.049	0.180	0.053	0.012	0.051					0.172	0.054	0.116	0.052
all - mR	27325	18	0.000	-0.144	0.154	0.026	0.071	-0.212	0.090	-0.006	0.053	0.149	0.060	0.012	0.053					0.155	0.057	0.140	0.068
mT, cvR	27326	19	0.000	-0.324	0.154	0.083	0.058																
mT, sT, cvR	27327	19	0.000	-0.308	0.127	0.081	0.061					0.086	0.052	0.011	0.053					0.093	0.042	0.093	0.042
mT, aT, cvR	27329	22	0.000	-0.297	0.134	0.084	0.064	-0.088	0.079	0.039	0.050									0.124	0.052	0.161	0.063
aT, cvR	27334	27	0.000					-0.203	0.070	0.044	0.047									0.163	0.049	0.090	0.050
sT, cvR	27336	29	0.000									0.123	0.050	-0.007	0.050					0.054	0.045	0.132	0.052
mT, sT	27336	29	0.000	-0.077	0.105	-0.021	0.050					0.124	0.049	-0.085	0.045								
mT, aT, sT	27337	30	0.000	0.000	0.116	-0.046	0.053	-0.112	0.072	-0.038	0.052	0.184	0.060	-0.095	0.046								
aT, sT	27338	31	0.000					-0.161	0.063	-0.013	0.050	0.186	0.054	-0.082	0.045								
cvR	27339	32	0.000																	0.078	0.039	0.102	0.050
mT	27340	33	0.000	-0.151	0.101	0.017	0.048																
sT	27340	33	0.000									0.126	0.047	-0.062	0.043								
mT, aT	27344	37	0.000	-0.150	0.103	0.017	0.049	0.000	0.063	-0.003	0.047												
.	27344	37	0.000																				
aT	27347	39	0.000					-0.065	0.057	0.021	0.045												

Table S2

Statistical summary of the models linking recruitment to climate metrics.

The ‘model’ column provides a short model description with: ‘T, R’ representing temperature and rainfall respectively; ‘m, a, s, cv’ the mean, amplitude of seasonal change, standard deviation and coefficient of variation (we used standardised variables). The notation ‘.’ highlights the model with no climatic variables, while ‘all’ highlights the model with all climatic variables included (for simplicity, the model labelled ‘all –mT’ presents the model including all variables but the mean temperature and its quadratic component).

In the 3 next columns, the support for each model is presented, based on Akaike criterion and weight.

The furthest right columns on the table present the coefficient for each model linking the recruitment to specific climatic variables (we present the estimate for the linear and quadratic effect together with their standard error).

Based on Akaike weight (w_i), all 16 first models include the mean rainfall and its quadratic component highlighting the importance of this parameter for badger recruitment (see table 2 of the main text).
