# Supplementary material for: A Multi-Metric Approach to Investigate the Effects of Weather Conditions on the Demographic of a Terrestrial Mammal, the European Badger (Meles meles)
Source: PLoS One. 2013 Jul 11;8(7):e68116. doi: 10.1371/journal.pone.0068116 (PMC3708947; doi:10.1371/journal.pone.0068116)
Supplement: Text S1 — In this appendix, we provide analyses of correlation between weather metrics presented in the main text. (PDF) [file pone.0068116.s003.pdf]

Text S1

In this appendix we provide analyses of correlation between weather metrics presented in the main text.

No correlation was found between the mean temperature and either the amplitude (b = 0.40 with F_{126} = 0.16, p = 0.84, R^2<0.01) or residual standard deviation in temperature (b = 0.08 with F_{126} = 1.0, p = 0.34, R^2<0.01) (Fig. S1-d and e). A positive correlation was found between the amplitude and the residual standard deviation in temperature (b = 0.49 with F_{126} = 39.8, p < 0.001, R^2 = 0.24) (Fig. S1-f).

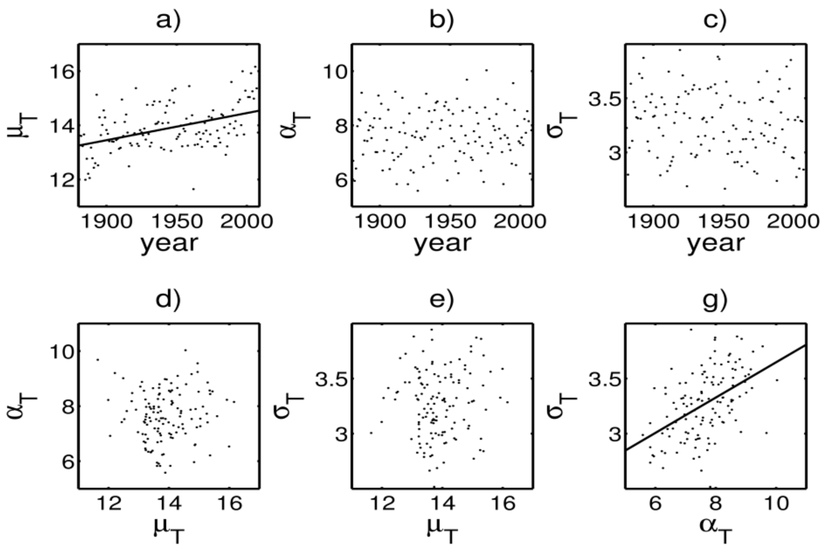

Figure S1: Mean (a), amplitude of seasonal changes (b) and residual standard deviation (c) in daily temperature for each year between 1881 and 2008. Only average temperature, μ_T, showed a significant long-term increasing trend. Plots (d-f) show the relationship between mean temperature and amplitude (d); between mean temperature and residual standard deviation (e); and a significant correlation between amplitude and residual standard deviation (f).

A negative correlation was also observed between the two metrics (b = -0.49 with F_{20} = 6.18, p = 0.02, R^2 = 0.24) (Fig. S2-c), that is, as mean rainfall increased the scaled variation in rainfall declined – suggesting that these circumstances correspond with persistently wetter conditions.

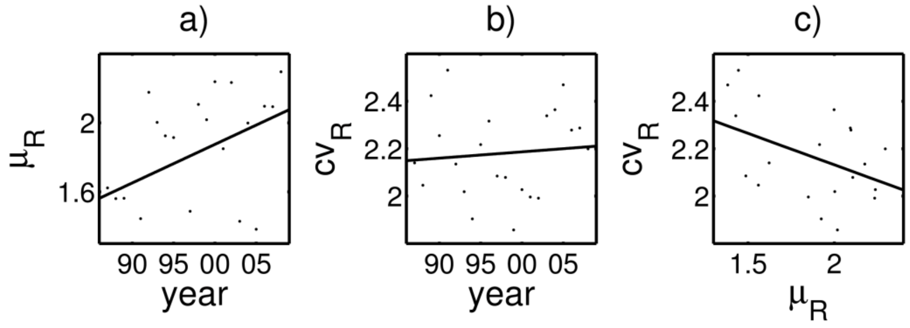

Figure S2: Mean (a) and coefficient of variation (b) in daily rainfall between April and September of each year between 1895 and 2009. Plot (c) shows the relationship between the mean and coefficient of variation. We observe that mean daily rainfall increases significantly with years, and a negative correlation between the mean and coefficient of variation.
